# Trends in incidence, prevalence, and disability-adjusted life years of schizophrenia in China from 1990 to 2021, with projections for 2022-2050

**DOI:** 10.3389/fpsyt.2025.1651350

**Published:** 2025-09-04

**Authors:** Jiawen Huo, Rui Li, Xuan Ren, Shuyi Zhu, Xiangdi Hu, Qiqing Tan, Yanxin Xu, Jing Chen, Junjiao Ping, Jing Wan, Tingyun Jiang, Aoxiang Luo

**Affiliations:** ^1^ School of Nursing, Guangdong Pharmaceutical University, Guangzhou, China; ^2^ Department of Psychiatry, The Third People’s Hospital of Zhongshan, Zhongshan, China

**Keywords:** schizophrenia, disease burden, epidemiology, China, GBD

## Abstract

**Objective:**

This study assessed trends in schizophrenia (SCZ) burden in China 1990–2021 and projected future trends 2022-2050.

**Methods:**

We analyzed data from the GBD 2021 study, employed the GBD method to integrate epidemiological data on age-standardized prevalence rate (ASPR), age-standardized incidence rate (ASIR), and age standardized disability adjusted life years rate (ASDR) to accurately assess the global burden of SCZ across various regions, genders, and age groups. Additionally, joint point regression analysis was applied to rigorously examine the time trends of anxiety disorders from 1990 to 2021, calculating the annual percentage change (APC), annual average percentage change (AAPC), and their corresponding 95% confidence intervals (CIs). Finally, a Bayesian age-period-cohort (BAPC) model was employed to predict the prevalence trends of SCZ from 2022 to 2050.

**Results:**

From 1990 to 2021, the ASPR and ASDR of SCZ in China increased steadily, with faster growth than global averages (ASPR AAPC: 0.130% vs. 0.021%; ASDR AAPC: 0.141% vs. 0.022%). In contrast, the ASIR remained stable in China (AAPC: 0.038%) but declined globally. Join point regression revealed an ASIR rebound after 2016 and an ASDR acceleration after 2016. In 2021, ASPR and ASIR peaked at ages 35–39 and 20-24, respectively, and burden growth was faster among females.

The BAPC model indicates that by 2050, the ASPR of SCZ in China is projected to reach 488.3 per 100,000 population (95% UI: 216.09-760.5), while the ASDR is expected to be 315.37 per 100,000 population (95% UI: 136.88-493.87).

**Conclusion:**

GBD 2021 data reveal a rising SCZ burden in China, especially in ASPR and ASDR, posing increasing public health challenges. Males bore a consistently higher burden, but the female burden increased faster than the global average, highlighting gender-specific concerns. Age patterns emphasize young and middle-aged populations as key targets for intervention. Projected increases in incidence and mortality call for enhanced, tailored prevention and treatment strategies, improved resource allocation, and strengthened mental health services to mitigate the societal impact.

## 1 Introduction

SCZ is a chronic, severe psychiatric disorder characterized by persistent disruptions in cognition, perception, emotion, and social functioning (1, 2). With a typical onset in late adolescence or early adulthood, it contributes to lifelong disability and is associated with high personal and societal costs (3). Globally, the lifetime prevalence of SCZ is approximately 1%, and individuals with the disorder often face significantly reduced life expectancy due to comorbid physical ill While they offer valuable foundational data for understanding the epidemiology ness and elevated suicide risk (4, 5). According to the GBD 2021, the DALY burden attributable to mental disorders was ranked 19th in 1990 and rose to 6th in 2021 (6), highlighting the global intensification of the mental health crisis. Descriptive epidemiological studies on the incidence, prevalence, and mortality of SCZ are essential for comprehensively understanding the current epidemiological landscape of the disorder and play a critical role in informing the planning and allocation of mental health services in public health systems (7). Although numerous studies have examined age-, sex-, and region-specific epidemiological characteristics of individuals with SCZ (8–11), most of these investigations are based on global or Western populations, with limited consideration of China’s unique sociocultural and demographic context.

In China, several cross-sectional studies have shown the prevalence of SCZ at the provincial level using diagnostic tools such as the Structured Clinical Interview for DSM (SCID) or the Composite International Diagnostic Interview (CIDI) (12). Although the global burden of SCZ has been widely studied, China’s unique social structure and stage of development present distinct epidemiological patterns and intervention challenges. Long-standing socioeconomic and healthcare resource disparities between urban and rural areas in China are associated with significant differences in environmental exposure, accessibility to medical services, and treatment outcomes for patients with schizophrenia. Existing studies have reported that patients with schizophrenia in rural areas tend to have higher mortality rates and may be more vulnerable to external stressors such as climate change (13, 14). In addition, the processes of urbanization and population mobility have been linked to increased psychological stress, with social isolation and adaptation difficulties among migrant populations identified as important ecological correlates and risk factors associated with schizophrenia (15). Prior research using national-level data, such as the GBD Study, has laid important groundwork in characterizing SCZ burden in China (16). However, updated analyses after 2020 and long-term forecasts remain limited, highlighting the need for continued research to support future policy directions. While existing studies provide valuable data on SCZ epidemiology in China, there is a notable lack of studies using predictive modeling tailored to China’s unique socioeconomic and demographic features to estimate the future burden of SCZ. This gap hampers the formulation of forward-looking interventions and evidence-based mental heal specifically tailored to China’s socioeconomic and demographic context policies. Therefore, there is a pressing need for locally grounded research that integrates temporal trends, regional disparities, and disease burden forecasting to better inform mental health policy and service planning in China. To address the current limitations in SCZ epidemiological research in China, this study aims to conduct a comprehensive analysis based on data from the Global Burden of Disease (GBD) 2021 study. The GBD project, led by the Institute for Health Metrics and Evaluation (IHME), is a large-scale global epidemiological initiative designed to assess and compare the burden of diseases, injuries, and risk factors across countries and regions. The GBD provides authoritative data to support public health decision-making worldwide, and its multi-decade continuity enables robust long-term trend analysis.

Therefore, this study aimed to (1) assess the long-term trends of SCZ burden in China from 1990 to 2021 using GBD 2021 data (2); identify key temporal inflection points in burden indicators through Joinpoint regression analysis (3); compare these trends with global patterns by sex and age group; and (4) project future trends of ASIR, ASPR, and ASDR in China up to 2050 using the Bayesian age–period–cohort (BAPC) model. Our findings may inform precision public health strategies and resource allocation for SCZ prevention and management in China.

## 2 Methods

### 2.1 Data sources

This study utilized publicly available, anonymized data from the Global Burden of Disease (GBD) 2021 database, accessible at https://vizhub.healthdata.org/gbd-results/ (accessed on March 15, 2025). The GBD 2021 study provides a comprehensive and up-to-date assessment of 369 diseases and injuries, along with 88 risk factors, across 204 countries and territories. It incorporates the most recent epidemiological evidence and applies improved standardized methodologies to ensure consistency and global comparability in disease burden estimation (17). As no individual-level or identifiable human data were involved, ethical approval and informed consent were not required. As the analysis did not involve any individual-level or identifiable human data, ethical approval and informed consent were not required. We selected schizophrenia (ICD-10 codes F20-F29) as the cause of interest. Under the ‘Measure’ category, we chose ‘Prevalence’, ‘Incidence’, and ‘DALYs’; under ‘Metric’, both ‘Number’ and ‘Rate’ were selected. Data were stratified by sex (male, female, both), and by age group (10-95+ years). The raw data were downloaded in CSV format and subsequently cleaned and organized using Microsoft Excel. All analyses employed age-standardized rates (ASRs), standardized to the GBD world standard population (2021 revision) to account for variations in age distributions and enable valid comparisons over time, across regions, and among demographic subgroups. Age standardization was performed using R version 4.4.1. The processed and standardized dataset was then used for trend analyses and future projections, including join point regression and the Bayesian Age-Period-Cohort (BAPC) model.

### 2.2 Relevant definitions

This study used disability-adjusted life years (DALYs) as the core indicator of disease burden. DALYs are defined as the sum of years lived with disability (YLDs) and years of life lost (YLLs). Due to the lack of reliable mortality data for schizophrenia, DALYs in this study were equivalent to YLDs, which were estimated by multiplying the number of prevalent cases by the corresponding disability weights. To assess uncertainty, 500 draws were conducted, and final estimates were reported as the mean across all draws, with 95% uncertainty intervals (UIs) defined by the 2.5th and 97.5th percentiles.

To adjust for differences in age distribution across populations, age-standardized prevalence rate (ASPR), incidence rate (ASIR), and DALY rate (ASDR) were also calculated. ASPR indicates the number of existing cases per 100,000 population, reflecting overall disease burden; ASIR denotes the number of new cases per 100,000, reflecting incidence risk; and ASDR refers to the number of DALYs per 100,000 population, serving as a key metric of total health loss.

### 2.3 Statistical analysis

#### 2.3.1 Descriptive analysis

To comprehensively evaluate trends in the burden of schizophrenia in China and globally, two descriptive analytical methods were employed in this study: First, the join point regression model, developed by Kim et al. in 1998 (18), was applied to time-series data on ASPR and ASDR from 1990 to 2021. This model detects one or more “join points” in the data to divide the study period into distinct intervals, allowing linear trends to be fitted within each interval to better characterize period-specific changes (19). For each segment, the Annual Percent Change (APC) and the overall Average Annual Percent Change (AAPC) were calculated, along with their 95% confidence intervals (95% CI). A statistically increasing trend was defined by a lower CI bound above zero, a decreasing trend by an upper CI bound below zero, and a stable trend by a CI containing zero. The analysis was conducted using JoinPoint 5.4.0. Second, to intuitively illustrate the overall magnitude of change over the study period, the total rate of change from 1990 to 2021 was computed using the following formula:


Total rate of change=(a2021−a1990)a1990 x 100%

This indicator serves as a complementary descriptive metric and is not based on statistical modeling.

#### 2.3.2 Forecasting analysis

We employed the Bayesian Age-Period-Cohort (BAPC) model to forecast the epidemiological trends of schizophrenia from 2022 to 2050. The methodology involved categorizing the population into age groups of 10–39 years, 40–59 years, 60–79 years, and above 80 years. We calculated the Bayesian model based on three factors: age, period, and cohort, and applied the Bayesian formula to compute the hypothetical probability distributions associated with these three factors. By integrating prior information with sample data, we derived posterior distributions. The statistical computations were conducted using R version 4.4.1.

## 3 Results

### 3.1 Comparison of schizophrenia ASIR, ASPR, and ASDR: China and global

**Figure 1:**
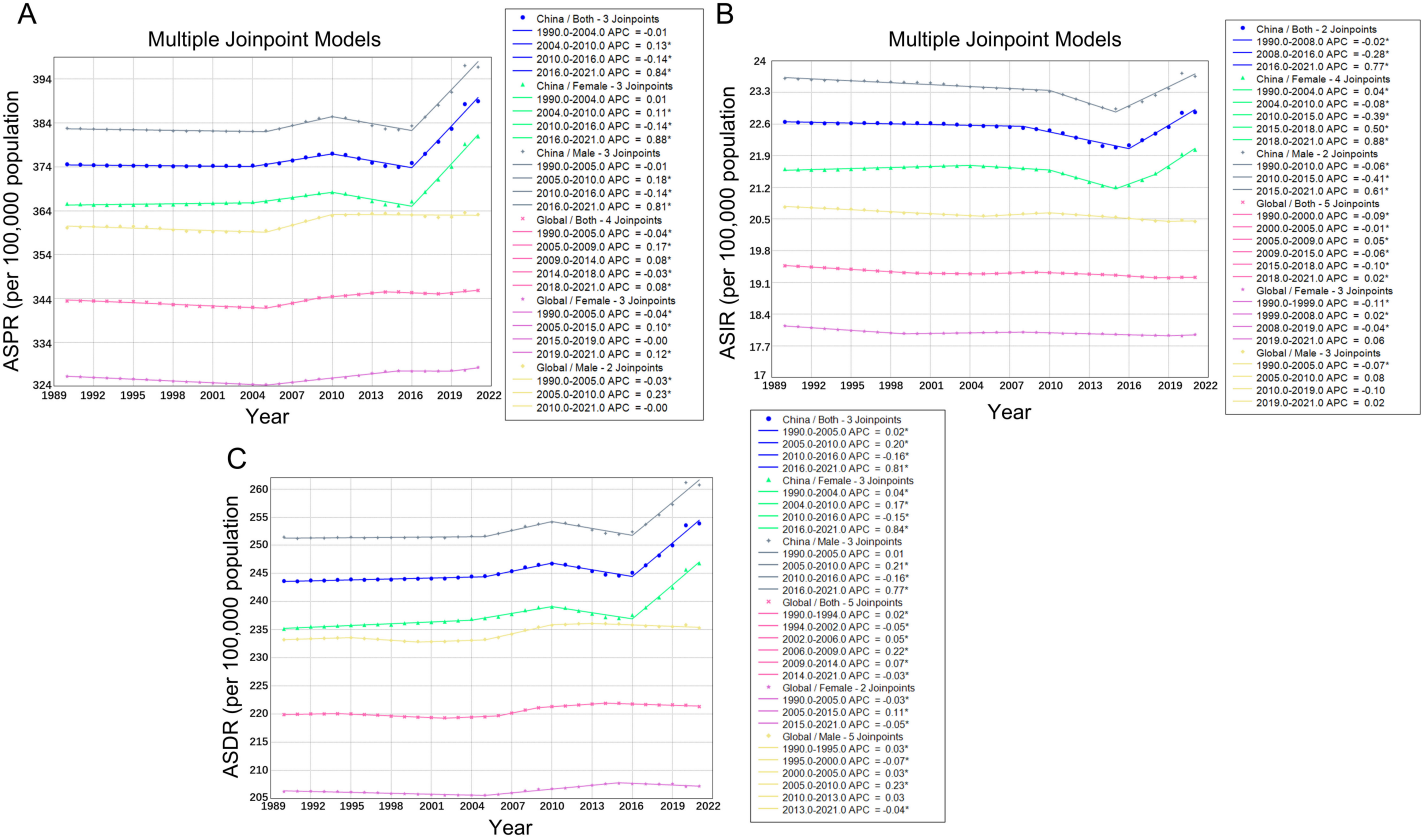
Trends in jointpoint regression analyses of SCZ across the global and China from 1990 to 2021: **(A)** ASPR of SCZ, **(B)** ASIR of SCZ, **(C)** ASDR of SCZ.

In China, the ASPR increased from 374.60 per 100,000 (95% UI: 306.46-449.58) in 1990 to 388.99 per 100,000 (95% UI: 318.13-467.59) in 2021, representing a total change of 3.84% (**Table 1**). As illustrated in **Figure 1A**, the ASPR curve demonstrated a gradual upward trend, with a noticeable acceleration in slope occurring around 2016. This trend was further confirmed by join point regression, which yielded an AAPC of 0.130% (95% CI: 0.123%-0.138%, P< 0.001), with a slightly faster increase in females (AAPC = 0.139%) than in males (AAPC = 0.127%) (**Table 2**). In contrast, the global ASPR remained relatively stable over the same period, rising only from 343.43 to 345.84 per 100,000 (a 0.70% increase), with a minimal AAPC of 0.021%. The global trend line showed no evident inflection point, indicating a generally stable burden of SCZ worldwide (**Figure 1A**). The ASIR in China followed a classic U-shaped pattern—declining initially and then rising—where 2016 marked the lowest point and a critical turning year (**Figure 1B**). The ASIR was 22.65 per 100,000 (95% UI: 14.35-32.18) in 1990 and increased slightly to 22.86 per 100,000 (95% UI: 14.49-32.59) in 2021, with a total rate change of 0.95% (**Table 1**). The overall AAPC was 0.038% (95% CI: 0.029%-0.048%, P< 0.001), though sex-specific trends differed significantly: incidence among females showed a marked increase (AAPC = 0.072%, P< 0.001), whereas the trend in males remained nearly flat (AAPC = 0.011%, P = 0.008) (**Table 2**). In contrast, the global ASIR displayed a consistent downward trend, declining from 19.47 to 19.21 per 100,000, with an AAPC of -0.043% (-0.050% in males, -0.035% in females), and no inflection was observed around 2016. As for ASDR, China exhibited a steady upward trajectory, with acceleration becoming more prominent after 2016 and particularly sharp following 2019 (**Figure 1C**). The ASDR increased from 243.66 per 100,000 (95% UI: 172.75-318.19) in 1990 to 253.89 per 100,000 (95% UI: 179.57-333.92) in 2021, representing a total change of 4.20% (**Table 1**). The overall AAPC was 0.141%, with a higher rate in females (AAPC = 0.158%) than in males (AAPC = 0.130%), both of which were statistically significant (**Table 2**). In contrast, the global ASDR remained nearly unchanged over the same period, rising only slightly from 219.93 to 221.35 per 100,000, with an overall AAPC of just 0.022%, and no clear turning point was observed (**Figure 1C**).

**Table 1:** Burden of SCZ in the global and China from 1990 to 2021.

Indicator	Region	Gender	1990	2021	Total rate of change (%)
ASPR (per 100,000) (95% UI)	China	Both	374.60 (306.46,449.58)	388.99 (318.13,467.59)	3.84 (3.81, 4.01)
		Male	382.81 (312.52,460.28)	396.75 (324.26,476.57)	3.64 (3.76, 3.54)
		Female	365.60 (299.57,439.47)	380.95 (311.57,457.66)	4.20 (4.01, 4.14)
	Global	Both	343.43 (271.27,426.06)	345.84 (271.31,431.6)	0.70 (0.01, 1.30)
		Male	360.12 (284.18,446.88)	363.14 (284.83,453.47)	0.84 (0.23, 1.47)
		Female	326.24 (257.94,404.44)	328.29 (257.66,409.48)	0.63 (-0.11, 1.25)
ASDR (per 100,000) (95% UI)	China	Both	243.66 (172.75,318.19)	253.89 (179.57,333.92)	4.20 (3.95, 4.95)
		Male	251.46 (178.08,329.93)	260.72 (183.97,343.7)	3.68 (3.31, 4.18)
		Female	235.17 (166.67,308.7)	246.81 (174.69,323.68)	4.95 (4.81, 4.85)
	Global	Both	219.93 (153.99,292.83)	221.35 (154.31,296.15)	0.64 (0.21, 1.13)
		Male	233.24 (162.86,311.46)	235.31 (164.04,314.93)	0.89 (0.72, 1.11)
		Female	206.27 (144.19,275.15)	207.22 (144.21,277.63)	0.46 (0.01, 0.90)
ASIR (per 100,000) (95% UI)	China	Both	22.65 (14.35,32.18)	22.86 (14.49,32.59)	0.95 (0.96, 1.26)
		Male	23.61 (14.98,33.5)	23.65 (15.02,33.73)	0.19 (0.25, 0.68)
		Female	21.61 (13.61,30.83)	22.03 (13.73,31.51)	1.98 (0.95, 2.20)
	Global	Both	19.47 (11.7,28.88)	19.21 (11.41,28.77)	-1.31 (-2.5, -0.39)
		Male	20.77 (12.51,30.72)	20.45 (12.2,30.54)	-1.54 (-2.47, -0.61)
		Female	18.14 (10.88,27.02)	17.95 (10.59,26.97)	-1.07 (-2.66, -0.19)

**Table 2:** Trend analysis of the SCZ burden in the global and China from 1990 to 2021, AAPC–Average annual percent change.

Indicator	China			Global		
	Male	Female	Both	Male	Female	Both
Prevalence						
AAPC	0.127	0.139	0.130	0.022	0.019	0.021
Lower CI	0.118	0.133	0.123	0.017	0.017	0.018
Upper CI	0.137	0.145	0.138	0.026	0.021	0.023
P-value	<0.001	<0.001	<0.001	<0.001	<0.001	<0.001
Incidence						
AAPC	0.011	0.072	0.038	-0.050	-0.035	-0.043
Lower CI	0.003	0.065	0.029	-0.055	-0.038	-0.045
Upper CI	0.022	0.079	0.048	-0.047	-0.033	-0.042
P-value	0.008	<0.001	<0.001	<0.001	<0.001	<0.001
Disability-adjusted life years						
AAPC	0.130	0.158	0.141	0.030	0.013	0.022
Lower CI	0.121	0.152	0.135	0.027	0.010	0.020
Upper CI	0.140	0.164	0.149	0.033	0.016	0.024
P-value	<0.001	<0.001	<0.001	<0.001	<0.001	<0.001

### 3.2 Sex-specific analysis of schizophrenia burden in China and globally in 2021

**Figure 2:**
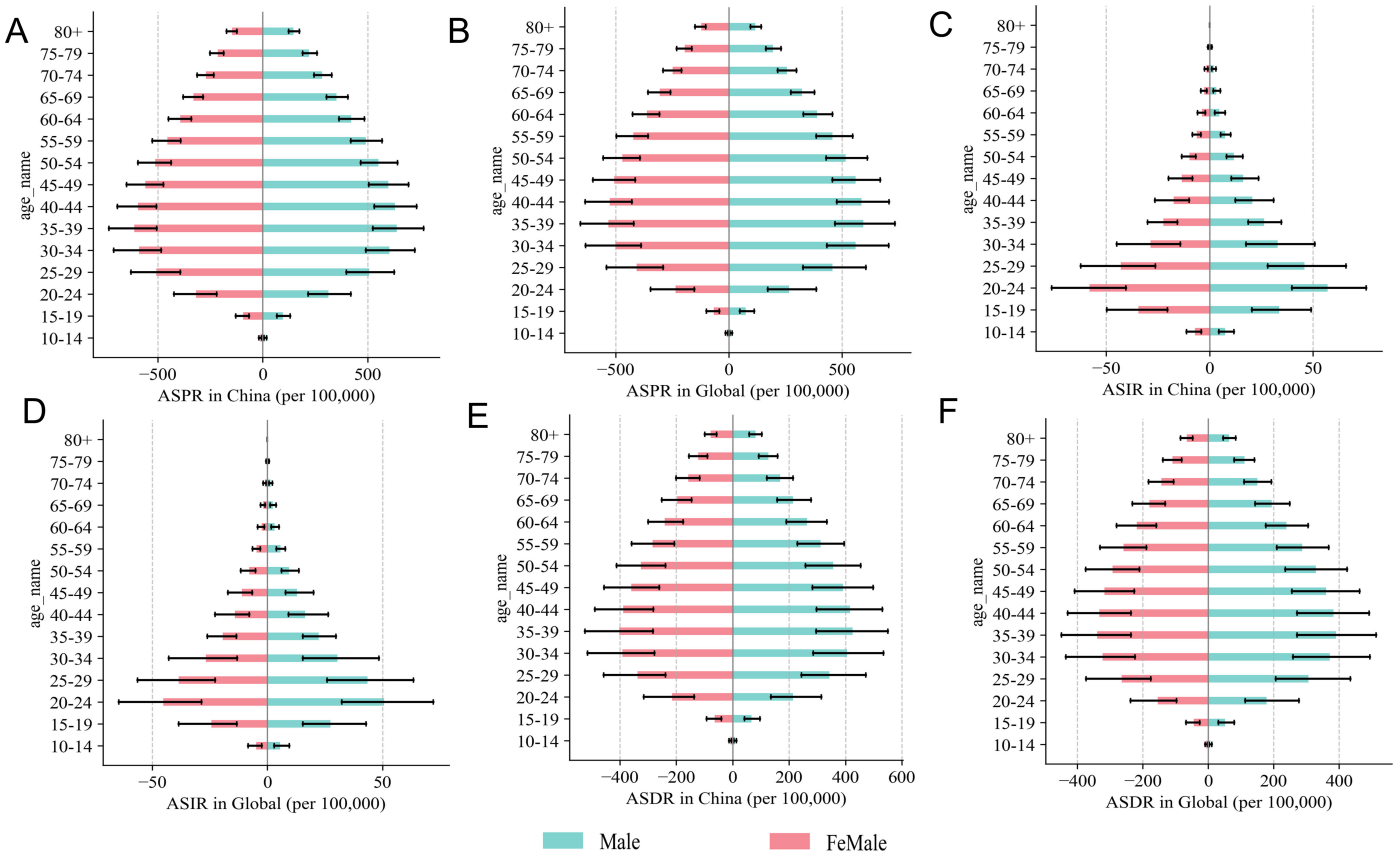
Sex-specific differences across age groups in age-standardized prevalence, incidence, and DALY rates of SCZ in China and Global in 2021. **(A)** ASPR in China, **(B)** ASPR in Global, **(C)** ASIR in China, **(D)** ASDR in Global, **(E)** ASDR in China, **(F)** ASDR in Global.

**Figure 3:**
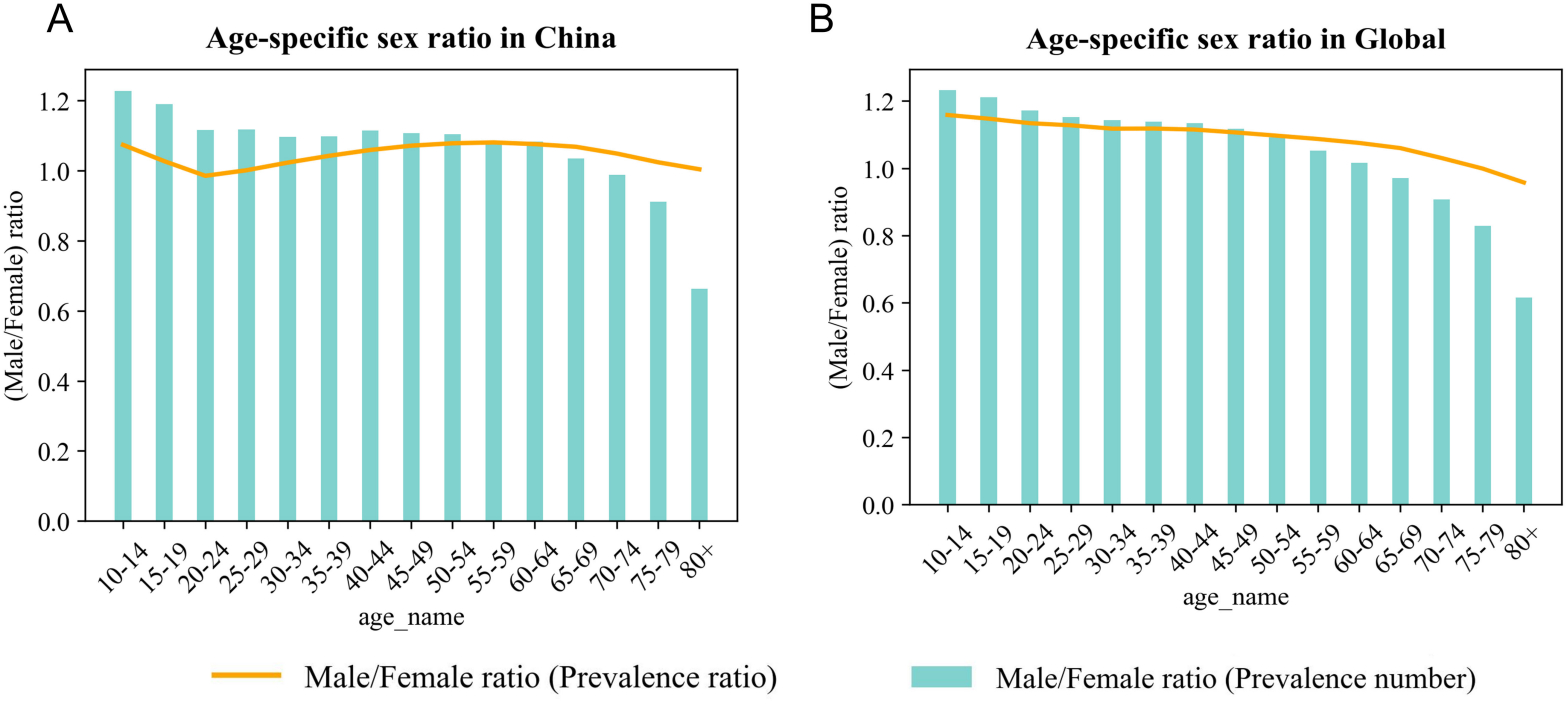
Age-specific sex ratio of SCZ prevalence in China and Global in 2021: **(A)** Age-specific sex ratio in China, **(B)** Age-specific sex ratio in Global.

In 2021, the ASPR of SCZ exhibited a characteristic bell-shaped distribution across age groups in both China and at the global level (**Figures 2A, B**). The burden peaked during young to middle adulthood, particularly between the ages of 20 and 44, and gradually declined with advancing age. The ASIR followed a funnel-shaped pattern in both settings (**Figures 2C, D**), with the highest incidence observed in the 20–24 age group, followed by a consistent decline in older age groups. Similarly, the ASDR mirrored the distribution of prevalence (**Figures 2E, F**), with the maximum burden concentrated among young and middle-aged adults. **Figure 3** further illustrates the sex-specific characteristics of SCZ prevalence across age groups by depicting the male-to-female prevalence ratio. In China, the ratio showed a biphasic pattern: it peaked at approximately 1.22 in the 10–14 age group, declined sharply to around 1.08 in the 20–24 age group, and rose again to a secondary peak of 1.15 in the 60–64 age group (**Figure 3A**). Thereafter, the ratio steadily decreased with age, falling below 1.00 among individuals aged 80 years and older. In contrast, the global trend displayed a monotonically decreasing pattern, starting at around 1.20 in the 10–14 age group and progressively declining to below 0.95 in those aged 80 and above (**Figure 3B**).

### 3.3 Trend analysis of sex-specific schizophrenia burden in China and globally from 1990 to 2021

**Figure 4:**
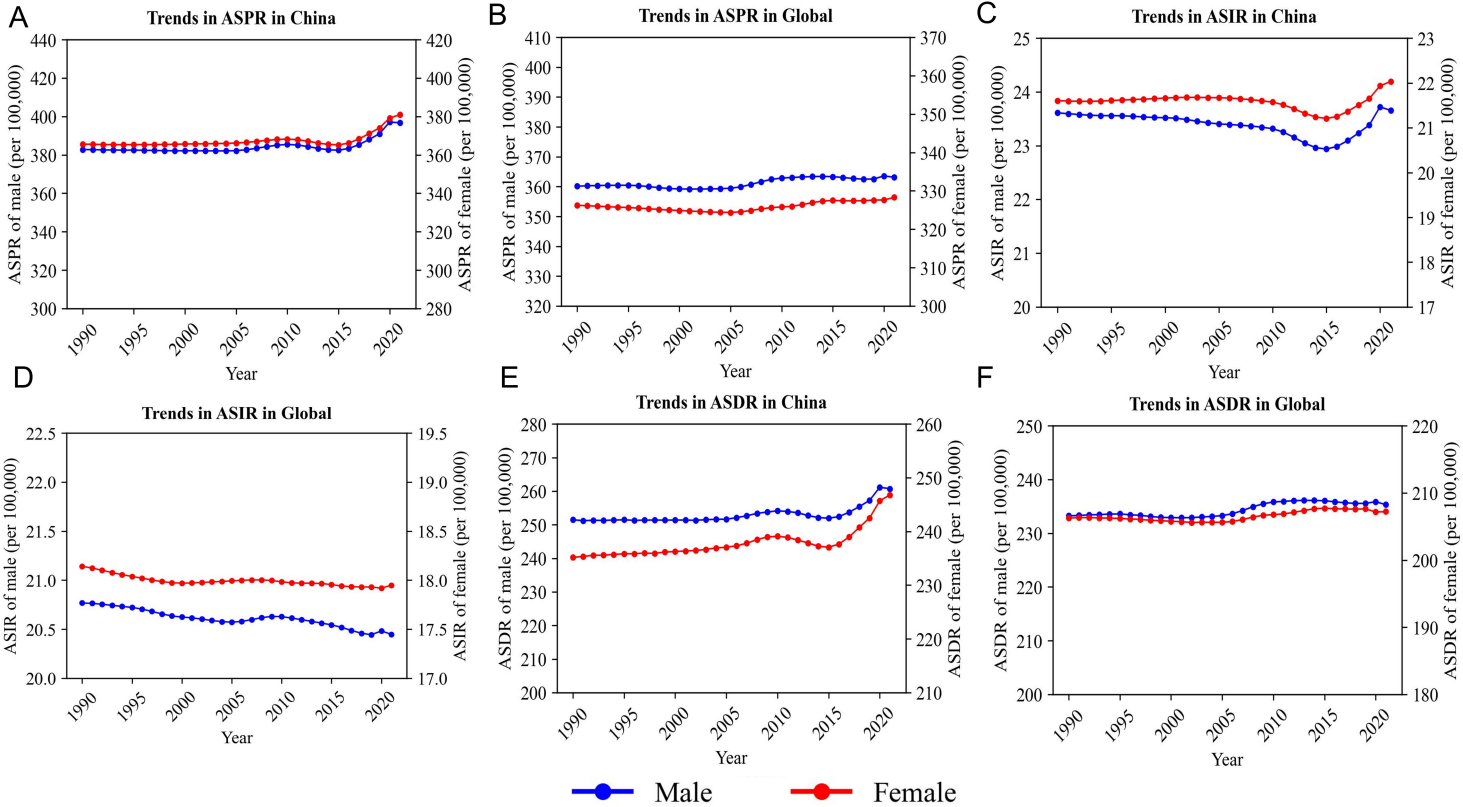
Trend analysis of sex-specific SCZ burden in China and globally from 1990 to 2021. **(A)** Trends in ASPR in China, **(B)** Trends in ASPR Global, **(C)** Trends in ASIR China, **(D)** Trends in ASIR in Global, **(E)** Trends in ASDR in China, **(F)** Trends in ASDR Global.

In China, the ASPR remained relatively stable for both males and females before 2015. However, following 2015, a gradual upward trend emerged in both sexes (**Figure 4A**). By 2021, the ASPR had exceeded 320 per 100,000 in males and 300 per 100,000 in females, with the sex gap widening progressively over time. In contrast, the global ASPR remained largely unchanged during the same period, showing only minor fluctuations and a relatively stable sex difference (**Figure 4B**).

From 1990 to approximately 2015, the ASIR in China showed a declining trend in both sexes, followed by a slight rebound. By 2021, the ASIR had reached approximately 19.4 per 100,000 in males and 17.8 per 100,000 in females, partially reversing the earlier decline (**Figure 4C**). At the global level, however, the ASIR continued to decline steadily across the entire period in both sexes, with no evidence of reversal or convergence. The sex gap remained consistent, contrasting sharply with the dynamic trajectory observed in China (**Figure 4D**).

Regarding the ASDR, trends in China were relatively flat before 2015, followed by a marked increase, particularly after 2019, when both male and female curves exhibited accelerated growth (**Figure 4E**). In comparison, the global ASDR curve showed only a slight increase throughout the same period, with no evident inflection points or pronounced sex differences (**Figure 4F**).

### 3.4 Projection of schizophrenia burden in China and globally from 2022 to 2050

#### 3.4.1 BAPC model performance

Utilizing the ASPR and ASDR data from China and globally between 1990 and 2011 as the training dataset, and the data spanning from 2012 to 2021 as the testing dataset, the fitting efficacy of the BAPC model was assessed. The findings demonstrated minimal values for the MSE, MAE, and MAPE, coupled with a fitting accuracy exceeding 98%, indicating a robust fit of the BAPC model (**Table 3**).

**Table 3:** BAPC model prediction fits for the burden of disease attributable to SCZ in China and globally from 1990 to 2021.

Indicator	Region	Gender	MSE	MAE	MAPE (%)	Fit accuracy (%)
ASPR	China	Both	32.11	4.27	1.11	98.89
		Male	29.04	4.08	1.04	98.96
		Female	36.34	4.45	1.19	98.81
	Global	Both	3.52	1.75	0.51	99.49
		Male	7.57	2.53	0.70	99.3
		Female	0.41	0.58	0.18	99.82
ASDR	China	Both	11.19	2.62	1.05	98.95
		Male	9.39	2.48	0.97	99.03
		Female	13.46	2.75	1.13	98.87
	Global	Both	2.01	1.28	0.58	99.42
		Male	3.96	1.80	0.76	99.24
		Female	0.40	0.53	0.26	99.74

#### 3.4.2 Disease burden of schizophrenia in China and globally from 2022 to 2050.

**Figure 5:**
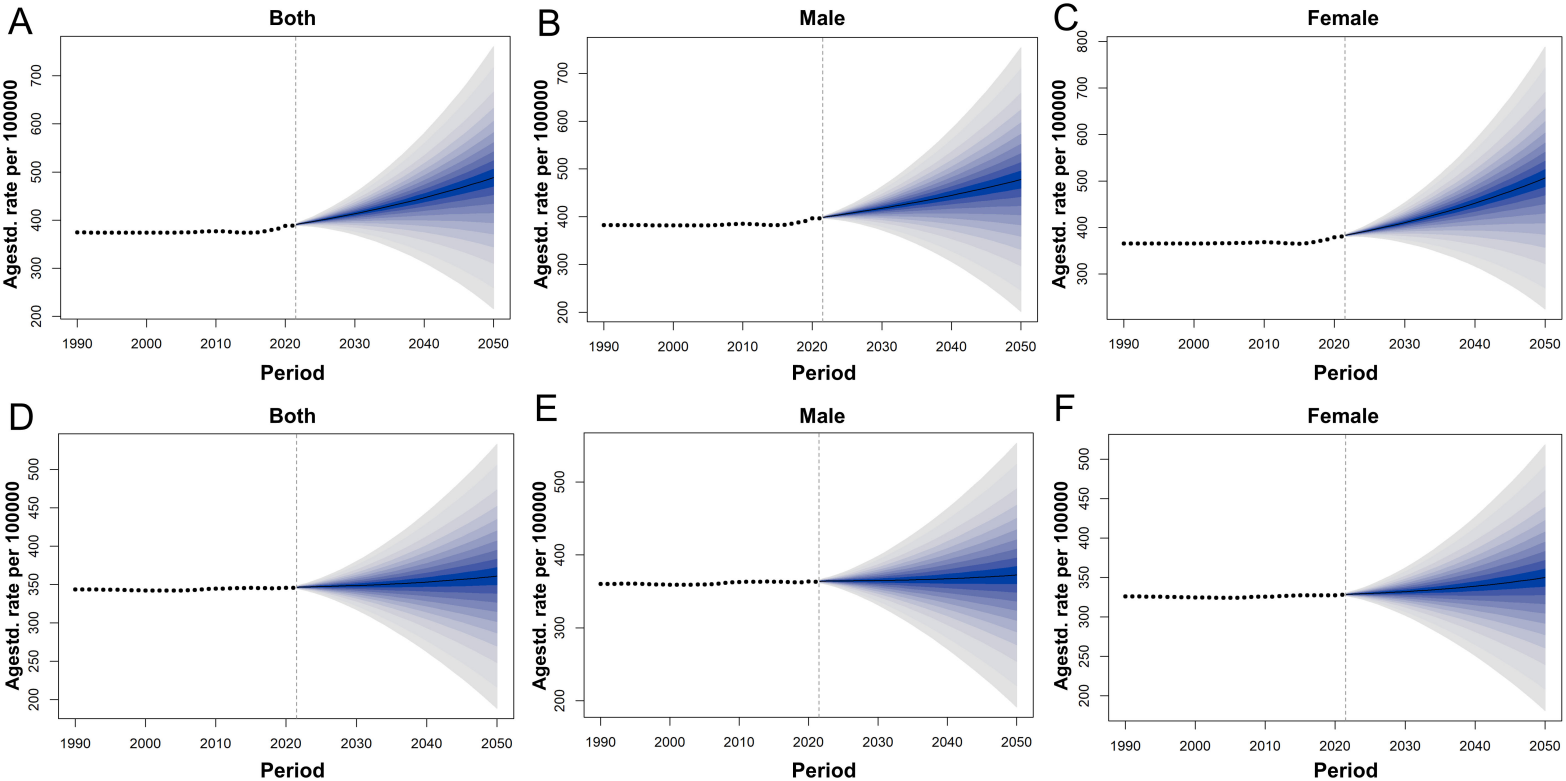
Actual and forecasted trends of ASPR for SCZ in China and Globally from 1990 to 2050: **(A)** ASPR of both sexes in China, **(B)** ASPR of males in China, **(C)** ASPR of females in China, **(D)** ASPR of both sexes in Global, **(E)** ASPR of males in Global, **(F)** ASPR of females in Global.

**Figure 6:**
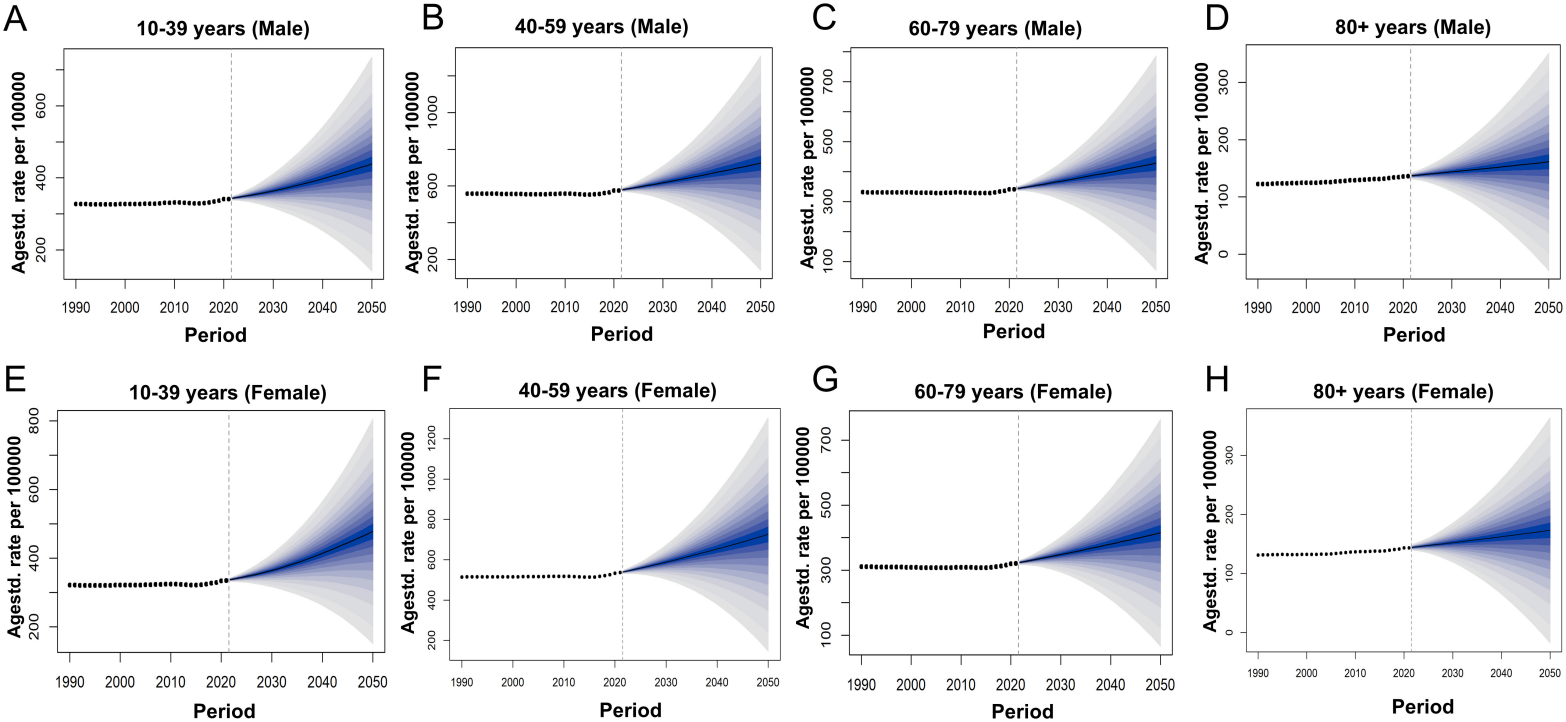
Actual and forecasted trends of ASPR for SCZ in China from 1990 to 2050: **(A)** ASPR of males in China aged 10 to 39, **(B)** ASPR of males in China aged 40 to 59, **(C)** ASPR of males in China aged 60 to 79. **(D)** ASPR of males in China aged above 80. **(E)** ASPR of females in China aged 10 to 39, **(F)** ASPR of females in China aged 40 to 59, **(G)** ASPR of females in China aged 60 to 79, **(H)** ASPR of females in China aged above 80.

**Figure 7:**
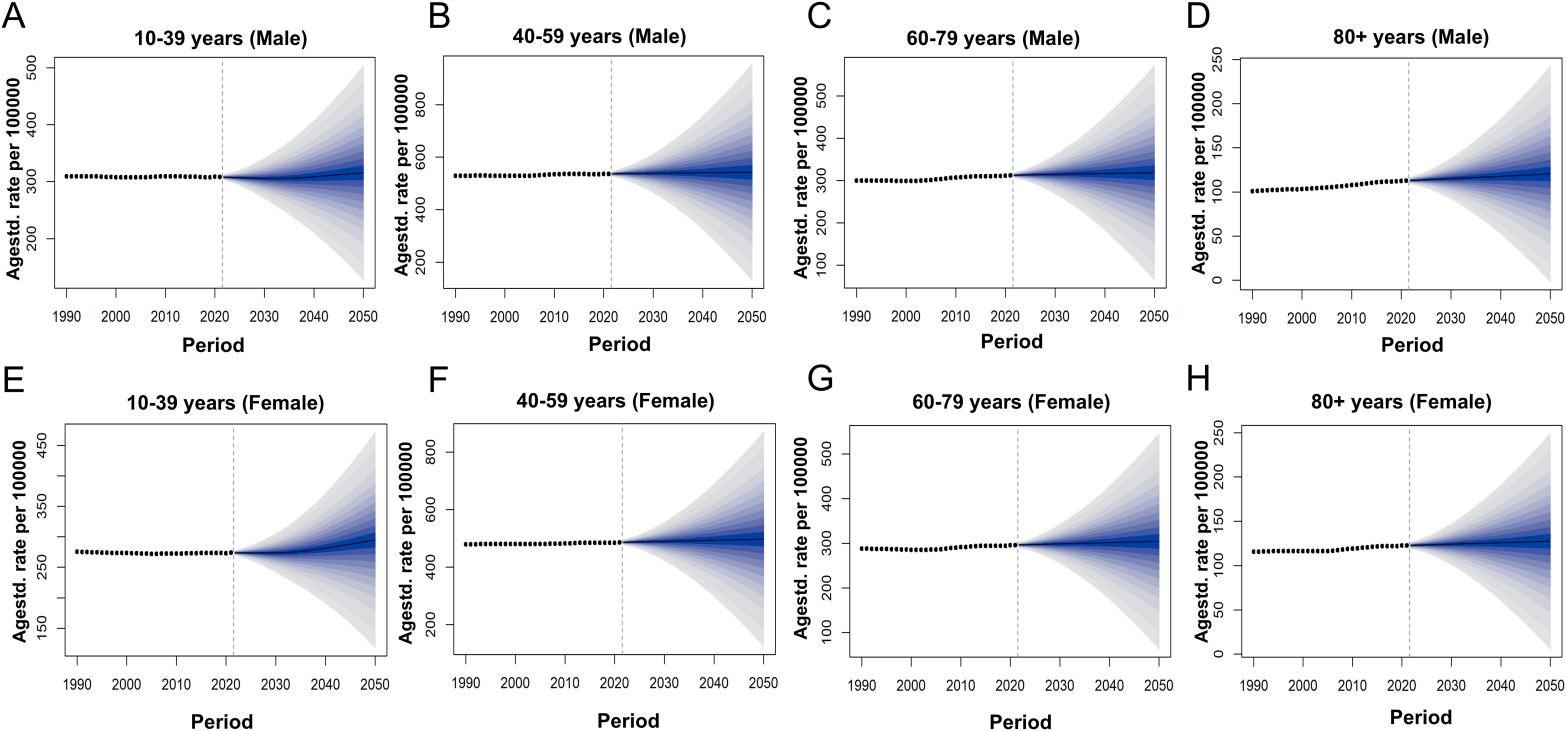
Actual and Forecasted Trends of ASPR for SCZ in Global from 1990 to 2050; **(A)** ASPR of males in Global aged 10 to 39, **(B)** ASPR of males in Global aged 40 to 59, **(C)** ASPR of males in Global aged 60 to 79 **(D)** ASPR of males in Global aged above 80. **(E)** ASPR of females in Global aged 10 to 39. **(F)** ASPR of females in Global aged 40 to 59, **(G)** ASPR of females in Global aged 60 to 79, **(H)** ASPR of females in Global aged above 80.

From 2022 to 2050, the ASPR and ASDR of SCZ in China are projected to increase steadily, whereas the global trends are expected to remain relatively stable (**Figure 5**). By 2030, the prevalence of SCZ in China among the total population is projected to reach 413.45 per 100,000 (95% UI: 370.16-456.74). By 2050, this figure is expected to increase to 488.3 per 100,000 (95% UI: 216.09-760.5). The ASDR is projected to rise from 269.37 per 100,000 population in 2030 (95% UI: 240.69-298.04) to 315.37 per 100,000 in 2050 (95% UI: 136.88-493.87). The rate of increase becomes more pronounced after 2030. In 2030, the highest projected prevalence of schizophrenia in China is observed among males aged 40–59 years, with a mean prevalence of 617.69 per 100,000 population (95% UI: 527.46-707.92). This indicates that middle-aged men will bear the greatest disease burden during this period. By 2050, the burden is projected to shift, with females aged 40-59 years exhibiting the highest prevalence, reaching 725.8 per 100,000 population (95% UI: 147.28-1304.33) (**Supplementary Table S1**, **Figure 6**). This trend suggests that in China, the disease burden among middle-aged women is continuously increasing, surpassing that of their male counterparts and highlighting a widening gender disparity in SCZ prevalence over time, whereas global projections indicate that the disease burden across sexes and age groups is expected to remain relatively stable (**Figure 7**).

## 4 Discussion

This study provides a comprehensive and systematic evaluation of the temporal trends in the burden of SCZ in China from 1990 to 2021, with projections extending into the 2050. While the ASIR in China has remained largely stable over the past three decades, both the ASPR and ASDR have shown persistent upward trends, reflecting the chronic and disabling nature of SCZ. Importantly, these increases are not evenly distributed across populations: females exhibited faster growth in both ASPR and ASDR than males, and this divergence is projected to widen in the coming decades. Furthermore, the burden of SCZ is increasingly concentrated in midlife adults-especially those aged 40–59 years-indicating a demographic shift in disease impact. These findings reveal the evolving landscape of SCZ burden in China and underscore the urgency of developing age- and gender-sensitive mental health policies.

From 1990 to 2021, the ASIR of SCZ in China remained relatively stable with a slight upward trend, contrasting with a global decline. Previous studies have shown that the global ASIR of SCZ may be related to SDI, with high-SDI countries generally experiencing increases in incidence and disease burden, while low-SDI countries tend to have stable or declining ASIR (9). As a high-middle SDI country, China fits this pattern in ASIR but stands out for rising ASPR and ASDR. This may result from improved case detection, reduced stigma, increased awareness, and better psychiatric infrastructure, especially in urban areas. Additionally, policy focus on severe mental illness may enhance surveillance and inflate the recorded burden (9, 15). The ASPR and ASDR showed sustained increases and are projected to rise further through 2050. This reflects the accumulating burden of SCZ as a chronic psychiatric disorder and the escalating challenges faced by long-term prevention and control efforts. The rising ASPR likely reflects better diagnosis and longer survival due to improved access to treatment and community-based care-yet these advances may not restore function (20). The continued increase in ASDR may also stem from treatment gaps and common comorbidities such as depression, substance use, and metabolic disorders, which worsen disability. Thus, stable incidence should not be seen as epidemiological progress when chronic symptoms and care disparities persist. India exhibits a relatively high prevalence of schizophrenia spectrum disorders but faces a significant treatment gap (approximately 72%), with socioeconomic factors such as low education levels and unemployment substantially increasing the disease burden (21). Brazil shows marked urban-rural differences in incidence rates, and insufficient mental health service coverage in rural areas exacerbates the disease burden (22). In contrast, China’s burden is more influenced by rapid urbanization and healthcare system reforms. These differences reflect the diversity in sociocultural contexts and healthcare resource allocation among LMICs, highlighting the need for policy interventions tailored to each country’s specific conditions.

Sex differences in the burden of SCZ are well-established and reflected in global and national disease burden data. Males exhibit higher incidence, mortality, and DALYs than females (5, 8, 23). This study validates prior observations and confirms the continuing sex-specific differences in SCZ burden. Notably, the rate of increase in the ASPR and ASDR among Chinese females has not only exceeded that of males but also surpassed global averages, suggesting a widening sex-based disparity in disease burden. Emerging evidence suggests that the faster increase in SCZ-related prevalence and DALYs among Chinese females may reflect a combination of biological, diagnostic, and sociocultural factors. Estrogen is believed to provide neuroprotection by modulating dopamine pathways and inflammation, helping to alleviate psychotic symptoms (24). However, hormonal fluctuations, particularly declining estrogen levels during perimenopause and menopause, may exacerbate symptoms and raise relapse risk, leading to a higher disease burden in middle-aged women (25). Diagnostic criteria developed predominantly from male samples may overlook female-specific features, such as later onset and greater emotional symptoms, contributing to delayed or missed diagnoses (26–28). Additionally, chronic stressors like caregiving roles, gender-based discrimination, and stigma may hinder help-seeking and worsen outcomes (27, 29). Women also face unique challenges in pharmacological treatment, such as increased sensitivity to side effects and insufficient attention to reproductive health, which may impair adherence and elevate long-term disability (29, 30). These factors may jointly explain the widening sex gap in SCZ burden observed in China and highlight the need for more tailored prevention, diagnosis, and treatment strategies for women. Given our finding that DALYs increased more rapidly among females, we also propose gender-sensitive care strategies, such as mental health education campaigns designed to reduce stigma toward women with psychiatric illness, and family-based psychoeducation that acknowledges the unique caregiving and social roles women often hold in Chinese society. In summary, while males bear a higher absolute burden of schizophrenia, the faster rise in ASPR and ASDR among Chinese females—exceeding both male and global trends—suggests a widening sex disparity. This may reflect hormonal changes, under recognition of female-specific symptoms, greater psychosocial stress, and treatment challenges in women. These findings highlight the need for gender-sensitive mental health policies, including tailored screening, reproductive health support, and improved access to long-term psychosocial care for women, especially during midlife.

Analyzing SCZ patients by different age groups in 2021 revealed that the burden of SCZ in China exhibits a bimodal distribution, with incidence and prevalence peaking during late adolescence to early adulthood (approximately 15 to 40 years), which is considered a critical window for long-term disability burden (16, 31). The neurodevelopmental hypothesis posits that disruptions in brain maturation, such as aberrant synaptic pruning, cortical development, and dopaminergic regulation-which often originate prenatally or in early life, become manifest during adolescence and early adulthood (32–34). Despite growing evidence supporting these mechanisms, early detection and intervention remain insufficient in many countries, particularly within school and primary care settings (2). Therefore, future public health strategies should prioritize these high-risk age groups by implementing structured, school- and community-based screening programs. These should include age-specific mental health assessments-particularly targeting adolescents and young adults-and comprehensive interventions such as cognitive behavioral therapy, family psychoeducation, and enhanced community-based follow-up for individuals at elevated risk. Additionally, culturally sensitive anti-stigma campaigns and personalized pharmacological treatments should be integrated to help delay disease onset and improve long-term clinical outcomes (35, 36). This study also identified a second peak in the male-to-female prevalence ratio of SCZ in China in 2021 among the 60–64 age group. During adolescence, societal gender role expectations and pressures may influence the sensitivity and reporting of diagnoses, while in older adults, differences in social support systems and access to health screenings may further affect diagnosis rates and disease burden. Given the lack of direct biological evidence for this phenomenon, future research should integrate epidemiological, sociocultural, and healthcare system factors to further investigate the underlying causes of this biphasic pattern and its implications for the management of schizophrenia.

Join point analysis revealed a significant turning point in 2016 for both ASPR and ASDR in China, aligning with the implementation of the *National Mental Health Work Plan* (2015–2020). Between 2009 and 2015, the 686 Program, initiated in 2005, provided free medication and follow-up subsidies (37). The 2015 Mental Health Work Plan and the scaling-up of the 686 Program subsequently expanded community-based screening and improved service accessibility, especially in underserved regions (38, 39). Framed by the stress–vulnerability model, which posits that the onset of schizophrenia results from the interaction between individual vulnerability and chronic environmental stressors, these policy interventions alleviated key stressors-namely economic hardship, geographic barriers, and social stigma—thereby lowering individuals’ threshold for perceiving and reporting symptoms. Consequently, previously undetected cases emerged in the healthcare system. The 2016 inflection should therefore be interpreted as a systematic unmasking of latent cases rather than a true surge in schizophrenia incidence. As Vigo et al. noted, inadequate service coverage leads to substantial under-ascertainment of mental disorders; expanding detection capacity increases reported prevalence without necessarily indicating a rise in true incidence (40). Through the lens of the stress-vulnerability model, we can better understand how policy and societal factors, by modulating stress perception and help-seeking behavior, indirectly shape the spatiotemporal patterns of schizophrenia burden.

From 2019 onwards, the incidence of SCZ began to trend upward, a trend that may be associated with the emergence of the COVID-19 pandemic. Coronaviruses, including SARS-CoV-2, possess neurotropic characteristics that facilitate central nervous system invasion and provoke excessive cytokine release, thereby exacerbating psychiatric manifestations (41). Furthermore, psychosocial stressors induced by the pandemic interact synergistically with inherent neurobiological vulnerabilities, as postulated by the Diathesis-Stress Model (33). Specifically, dysregulation of the hypothalamic-pituitary-adrenal (HPA) axis increases stress sensitivity, resulting in cortisol-mediated dysregulation of dopaminergic and glutamatergic neurotransmission, processes that may precipitate or exacerbate psychotic disorders. Therefore, the pronounced increase in SCZ incidence observed after 2019 is likely attributable to the combined impact of COVID-19-associated neurobiological insults and pandemic-related psychosocial stressors, superimposed upon underlying neurodevelopmental susceptibilities. Beyond neurobiological mechanisms, pandemic-related psychosocial stressors—such as service disruptions, caregiving burdens, and social isolation-may have exacerbated schizophrenia burden, particularly among vulnerable groups (42–44). While the COVID-19 pandemic may be associated with changes in the incidence of schizophrenia, our join point regression analysis did not identify 2019 as a statistically significant breakpoint. This may reflect delayed effects of the pandemic on mental health service utilization and diagnostic reporting, or the possibility that data up to 2021 were insufficient to capture post-pandemic trend changes. Therefore, continued monitoring is warranted to assess the long-term impacts of COVID-19 on the burden of schizophrenia.

Our BAPC model predicts further rises in ASPR and ASDR through 2050, highlighting the urgent need for integrated, long-term mental health services. Specifically, the model forecasts that the disease burden among middle-aged adults will continue to increase after 2022, with the burden among females aged 40 to 59 years projected to surpass that of males in the same age group by 2050. Therefore, we advocate for the expansion of community-based mental health services, including assertive community treatment and mobile outreach teams, to address care continuity and accessibility, especially in underserved rural regions where treatment gaps remain substantial. Therefore, we recommend targeted measures such as piloting rural telepsychiatry services to improve specialist access in underserved areas, expanding community mental health outreach teams to ensure continuity of care, and integrating mental health care into primary health services to facilitate early detection and long-term management.

## 5 Limitations of the study

This study, drawing upon GBD 2021 data and established analytical frameworks, offers a comprehensive and timely assessment of the burden of schizophrenia in China. However, several limitations should be considered when interpreting these findings. First, the GBD 2021 database lacks provincial- and urban-rural stratification, preventing us from capturing regional heterogeneity across China’s vast territory (45). Second, as a secondary analysis, our estimates inherit GBD’s inherent uncertainties—especially under-reporting and variable surveillance quality—which may bias both historical trends and future projections (45). Third, the joinpoint and BAPC models use population-level data and cannot establish causality or account for individual-level risk factors; they also do not adjust for short-term disruptions such as the COVID-19 pandemic. Future work incorporating finer geographic and individual data is needed to refine these national-level trajectories (46). Finally, while this study highlights temporal and demographic trends, future research would benefit from incorporating more granular data on individual-level risk factors, healthcare access, and treatment outcomes.

## 6 Conclusion

This study, based on GBD 2021 data and BAPC modeling, provides a comprehensive projection of SCZ burden in China through 2050. While the ASIR is expected to remain stable, both ASPR and ASDR are projected to rise steadily, with the burden increasingly concentrated among midlife adults and notably among females-suggesting a widening gender disparity. The projected ASPR growth also exceeds global averages. These trends highlight the chronic, disabling nature of SCZ and underscore the urgent need for long-term, gender- and age-sensitive strategies. Women, particularly in midlife, may face greater vulnerability due to biological, psychosocial, and treatment-related factors. Policies should prioritize early detection, long-term rehabilitation, and community-based care tailored to female-specific needs. Integrating SCZ prevention into national public health programs-leveraging platforms such as primary care and school-based services-will be essential to improve early access and long-term outcomes. Sustained monitoring and data integration are critical to guide equitable mental health responses.

## Data Availability

The original contributions presented in the study are included in the article/**Supplementary Material**. Further inquiries can be directed to the corresponding author/s.
